# Thermal Profiles in Water Injection Wells: Reduction in the Systematic Error of Flow Measurements during the Transient Regime

**DOI:** 10.3390/s23239465

**Published:** 2023-11-28

**Authors:** German Alberto Echaiz Espinoza, Gabriel Pereira de Oliveira, Verivan Santos Lima, Diego Antonio de Moura Fonseca, Werbet Luiz Almeida da Silva, Carla Wilza Souza de Paula Maitelli, Elmer Rolando Llanos Villarreal, Andrés Ortiz Salazar

**Affiliations:** 1 Department of Electronics Engineering, Universidad Nacional de San Agustin de Arequipa, Arequipa 04002, Peru; gechaiz@unsa.edu.pe; 2 Department of Computer Engineering and Automation, Federal University of Rio Grande do Norte (DCA-UFRN), Natal 59072-970, RN, Brazil; gabrpereira20@gmail.com (G.P.d.O.); verivan@petrobras.com.br (V.S.L.); diegomoura@dca.ufrn.br (D.A.d.M.F.); werbethluizz@hotmail.com (W.L.A.d.S.); andres@dca.ufrn.br (A.O.S.); 3 Department of Petroleum Engineering, Federal University of Rio Grande do Norte (DPET-UFRN), Natal 59072-970, RN, Brazil; carla.maitelli@ufrn.br; 4 Department of Natural Sciences, Mathematics, and Statistics, Federal Rural University of Semi-Arid (DCME-UFERSA), Mossoró 59625-900, RN, Brazil

**Keywords:** oil reservoir, thermal profile, geothermal profile, flow rate injection

## Abstract

This article presents an analytical solution for calculating the flow rate in water injection wells based on the established thermal profile along the tubing. The intent is to minimize the intrinsic systematic error of classic quasi-static methodologies, which assume that all thermal transience on well completion has passed. When these techniques are applied during the initial hours of injection well operation, it can result in errors higher than 20%. To solve this limitation, the first law of thermodynamics was used to define a mathematical model and a thermal profile was established in the injection fluid, captured by using distributed temperature systems (DTSs) installed inside the tubing. The geothermal profile was also established naturally by a thermal source in the earth to determine the thermal gradient. A computational simulation of the injection well was developed to validate the mathematical solution. The simulation intended to generate the fluid’s thermal profile, for which data were not available for the desired time period. As a result, at the cost of greater complexity, the systematic error dropped to values below 1% in the first two hours of well operation, as seen throughout this document. The code was developed in Phyton, version 1.7.0., from Anaconda Navigator.

## 1. Introduction

When working with water injection in an oil production field, knowing the injected flow rate is crucial. Deviations from the designed flow rates can result in lost oil production and damage to the reservoir in the worst case. In the case of multiple injection zones, where fluid is injected simultaneously in several sections along the injection well, the quality of the flow rate value is more critical due to the risk of greater flow deviations between zones, where a problem in one injection zone can completely change the flow rate being injected in another zone.

Currently, the flow rate is measured at the surface near the well. Typically, an orifice plate takes the measurement [1]. Another method could be used. To measure the flow below the surface using well tubing, it is necessary to shut down the well to install and use specialized instrumentation (e.g., flow meters and radioactive tracers). The measurement is not continuous. It is executed at a single point in time, which makes these methods very expensive and not suitable for continuous well production. For wells with only one injection zone, this is sufficient. However, for wells with multiple zones, the proportion of the flow in each zone is estimated from the surface flow. The estimate is based on the pressure applied to mechanical flow regulators, which are installed in the injection zone. This approach normally leads to an incorrect injection flow rate.

To overcome these difficulties and limitations, several measurement techniques are being developed based on the thermal profile in the injection column obtained by so-called distributed temperature sensors (DTSs). In this technique, an enriched fiber at predetermined points provides, in real-time, the temperature along the production column through the intensity of the reflected light wave. From this thermal profile, it is possible to derive the flow rate transported inside the tube and, consequently, the injection flow [2].

As a result of the small radii of well completions (<1 m) compared to the radius of the formation, which is assumed to have infinite extension (>>1 m), all previous researchers have ignored the transient heat flow in the well completion (tubing, annulus, casing, and cementation). Ramey was one of them. He proposed the first practical and consistent work to predict the fluid’s thermal profile in the tubing. Since heat flow is a very slow process, the heat transient in well completion is still significant during the initial hours of well operation. This implies a significant systemic error inherent in the methodology.

Motivated by the need to reduce the systematic error in the calculation of the flow rate inside the tubing during the first hours of well operation and originally inspired by the work of Ramey, this paper proposes a solution that takes into account the effect of the transient heat flow in well completion on the prediction of the thermal profile of the fluid and, consequently, the flow rate, the desired variable.

## 2. Preliminaries

**Figure 1:**
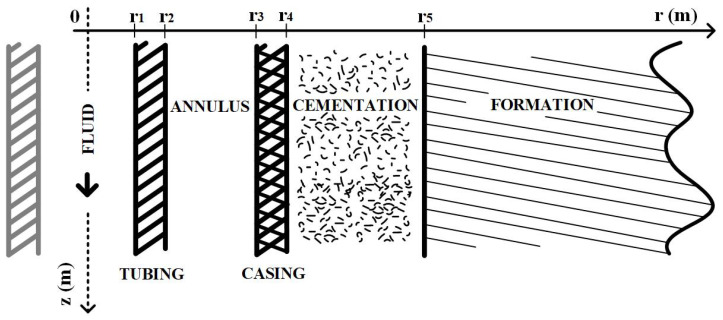
Standard well schematic.

Consider an injection well with the standard completion shown in Figure 1. Furthermore, assume that the well is in an idle state in thermal equilibrium with the formation. Under this condition, the temperatures of the well, completions, and formations are equal to the geothermal temperature, $T_{g}\left(z\right)$, which increases linearly with depth, given by:

(1) $$T_{g}\left(z\right)=az+b$$

where *z* is the vertical coordinate (depth), *a* is the geothermal gradient, and *b* is the surface temperature. The geothermal temperature is the temperature naturally established in the formation and wellbore by heat flow from the earth’s center. It is a very slow process compared to the thermal processes involved in well completion.

Assume that a new injection process starts at t=0. Furthermore, assume that the injection fluid has no phase change, is incompressible, and is colder than the reservoir. Under these conditions, as the fluid moves through the tubing, it gains thermal energy from its surroundings, and its temperature increases, creating a thermal profile along the tubing. The profile created is directly related to the flow rate in the tubing.

**Figure 2:**
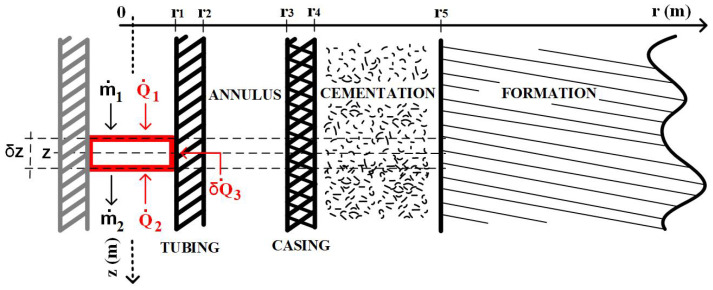
Control volumes and conservation of energy.

To calculate the flow rate for t>0, consider the application of the energy and mass conservation principle to the control volume (CV) highlighted in Figure 2. In this figure, $\dot{m}$ is the forced mass flow in the tubing, ${\dot{Q}}_{1}$, and ${\dot{Q}}_{2}$ are the heat flows entering the control volume by convection at the upper and lower surfaces, and $\delta {\dot{Q}}_{3}$ is the heat flow entering the control volume by convection at the inner tubing surface of size δz.

Assume that the flow rate is constant and the flow pattern is turbulent. As a result of this last characteristic, the fluid can be treated as an agglomerated system, in which the transience of the heat transfer in the fluid can be neglected because of the slowness of the transfer mechanisms in its environment. Consider also that the thermal properties are isotropic, homogeneous, and invariant with time and that there is no heat generation due to the fluid viscosity.

In this context, applying the principles of conservation of energy and mass to the CV, we have:

(2) $$\begin{array}{c} {\dot{E}}_{{\dot{m}}_{1}}\left(z-\frac{\delta z}{2},t\right)-{\dot{E}}_{{\dot{m}}_{2}}\left(z+\frac{\delta z}{2},t\right)+{\dot{Q}}_{1}\left(z-\frac{\delta z}{2},t\right)+{\dot{Q}}_{2}\left(z+\frac{\delta z}{2},t\right)+\delta {\dot{Q}}_{3}\left(r_{1},z,t\right)\\ =\frac{\partial E_{CV}\left(z,t\right)}{\partial t} \end{array}$$

where ${\dot{E}}_{\dot{m}}$ is the energy flow (thermal, kinetic, gravitational, and hydraulic potential energy) associated with the mass flow entering or leaving the control volume (CV). ${\dot{E}}_{\dot{C}V}$ is the total energy within the CV.

Since the vertical heat flows ${\dot{Q}}_{1}$ and ${\dot{Q}}_{2}$ are a negligible fraction of the heat transported by the mass flow, they are assumed to be zero in this paper. As a result, the mass flow inside the tubing is given by [3,4]:

(3) $$\begin{array}{c} \dot{m}≅\frac{2\pi r_{1}U\left(z,t\right)}{c}\left[T_{g}\left(z\right)-T_{f}\left(z,t\right)\right]/\frac{\partial T_{f}\left(z,t\right)}{\partial z}-\pi {\left(r_{1}\right)}^{2}\rho \frac{\partial T_{f}\left(z,t\right)}{\partial t}/\frac{\partial T_{f}\left(z,t\right)}{\partial z} \end{array}$$

where *c* is the specific thermal capacity of the fluid, ρ is the specific volume of the fluid, $T_{f}\left(z,t\right)$ is the temperature of the fluid at the center of tubing (r=0), and U(z,t) is the global heat transfer coefficient, with $r=r_{1}$ as the reference. The coefficient U(z,t) includes all thermal resistance to heat propagation, from convection at the inner surface of the tubing at $r=r_{1}$ to the radial limit of thermal disturbance caused by transporting the fluid at a colder temperature. For example, if the thermal disturbance is beyond the cementation, this coefficient includes the following resistances: tubing conduction, natural convection in the annulus, casing conduction, cementation conduction, and finally, formation conduction from $r=r_{5}$ to the thermal disturbance inside the formation. It is defined by [5]:

(4) $$\begin{array}{c} U\left(z,t\right)=\frac{\delta {\dot{Q}}_{3}\left(z,t\right)}{2\pi r_{1}\delta z\left[T_{g}\left(z\right)-T_{f}\left(z,t\right)\right]} \end{array}$$

The calculation of the flow rate, $\dot{m}$, as can be deduced from Equation (3), depends on the value of U(z,t), which in turn depends on the convection coefficient on the inner surface of the tubing, which depends on the desired flow rate. In other words, to determine the value of the flow rate, it is necessary to solve the following system of nonlinear equations:

(5) $$Pr=\frac{\mu c}{k}$$

(6) $$Re=\frac{\rho \upsilon_{med}\left(2r_{1}\right)}{\mu }$$

(7) $$f={\left[0.79\ln\left(Re\right)-1.64\right]}^{-2}$$

(8) $$h=\frac{(f/8)(Re-1000)Pr}{1+12.7{\left(f/8\right)}^{0.5}\left({Pr}^{2/3}-1\right)}\frac{k}{2r_{1}}$$

(9) $$\begin{array}{c} \tilde{U}≅U\left(\tilde{z},t\right) \end{array}$$

(10) $$\dot{m}≅\frac{2\pi r_{1}\tilde{U}}{c}\frac{\int_{z_{1}}^{z_{2}}\left[T_{g}\left(z\right)-T_{f}\left(z,t\right)\right]dz}{T_{f}\left(z_{2},t\right)-T_{f}\left(z_{1},t\right)}-\pi {\left(r_{1}\right)}^{2}\rho \frac{\int_{z_{1}}^{z_{2}}\left[\partial T_{f}\left(z,t\right)/\partial t\right]dz}{T_{f}\left(z_{2},t\right)-T_{f}\left(z_{1},t\right)}$$

where Pr is the Prandtl number, Re is the Reynolds number, *f* is the friction factor, *h* is the convection coefficient, $U(\tilde{z},t)$ is the global average heat transfer coefficient in the interval [$z_{1}$,$z_{2}$], and $\dot{m}$ is the desired mass flow rate. The variables μ, *c*, *k*, and ρ are the absolute viscosity, specific heat capacity, thermal conductivity, and specific mass, respectively, all relative to the fluid in flow.

Equation (7) corresponds to Petukhov’s first explicit equation for calculating the friction factor in smooth tubes. It is valid for 3 × $10^{3}<Re<5$ × $10^{6}$ [6].

Equation (8) is the Gnielinski equation for calculating the convection coefficient for a forced turbulent flow moving in a tube. It is valid for 0.5≤Pr≤2 × $10^{3}$ and 3 × $10^{3}<Re<5$ × $10^{6}$ [6].

**Figure 3:**
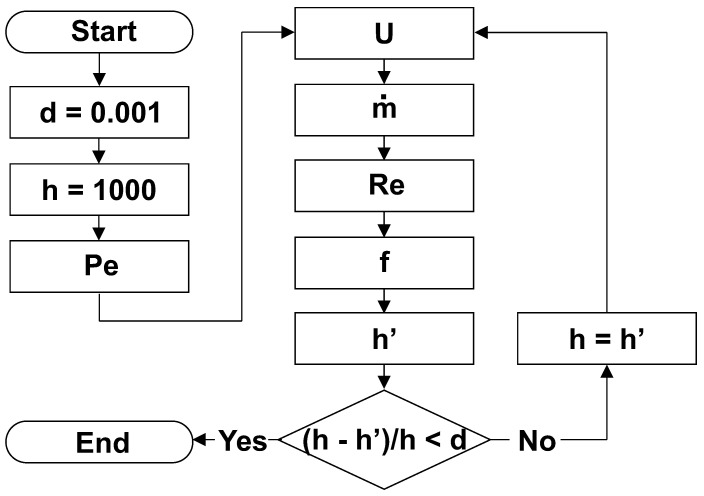
Algorithm for solving the equation system.

The system resulting from Equations (5) to (10) can be solved, among other methods in the literature, by executing the algorithm shown in Figure 3.

The approach chosen for calculating $\tilde{U}$ will define its applicability and the intensity of the corresponding systematic error. This article presents two approaches: (a) the quasi-static approach, which assumes that the thermal processes are already slow enough to be considered in thermal equilibrium, and (b) the purely analytical approach, which seeks the solution by solving the differential heat transfer equations.

## 3. Quasi-Static Approach

As a result of the small radius of the completion compared to the formation ($r>>r_{5}$), which corresponds to the radius of the outer cementation surface ($r=r_{5}$ in Figure 2), the quasi-static solutions ignore all thermal transients in the fluid and completion (tubing, annulus, casing, and cementing). As a result, still taking Figure 2 as a reference, the global heat transfer coefficient and the mass flow will be given by:

(11) $$\begin{array}{c} \tilde{U}≅{\left[\frac{1}{h}+\frac{r_{1}}{k_{tub}}\ln\left(\frac{r_{2}}{r_{1}}\right)+\frac{r_{1}}{r_{2}}\frac{1}{h_{comb}}+\frac{r_{1}}{k_{rev}}\ln\left(\frac{r_{4}}{r_{3}}\right)+\frac{r_{1}}{k_{cim}}\ln\left(\frac{r_{5}}{r_{4}}\right)+\frac{r_{1}f\left(t\right)}{k_{for}}\right]}^{-1} \end{array}$$

(12) $$\dot{m}≅\frac{2\pi r_{1}\tilde{U}}{c}\frac{\int_{z_{1}}^{z_{2}}\left[T_{g}\left(z\right)-T_{f}\left(z,t\right)\right]dz}{T_{f}\left(z_{2},t\right)-T_{f}\left(z_{1},t\right)}$$

where $k_{tub}$, $k_{rev}$, $k_{cim}$, and $k_{for}$, are the thermal conductivity of tubing, casing, cementation, and formation, respectively. $r_{1}$, $r_{2}$ are the tubing’s inner radius and external radius in m, respectively, $r_{3}$ and $r_{4}$ are the casing’s inner and outer radius, respectively, $r_{5}$ is the cementation’s external radius, $h_{comb}$ is the combined heat transfer coefficient, and f(t) is the transient function for heat propagation in the formation up to the thermal perturbation limit [5]. This function encompasses the entire time dependence of heat propagation in the formation. It is the primary variable to be determined in this methodology. It is defined by:

(13) $$\begin{array}{c} f\left(t\right)=\frac{2\pi k_{for}\left[T\left(r_{5},z,t\right)-T_{g}\left(z\right)\right]\Delta z}{\Delta \dot{Q}\left(r_{5},z,t\right)} \end{array}$$

In Equation (13), $T(r_{5},z,t)$ is the temperature at the interface with the formation, $T_{g}\left(z\right)$ is the geothermal temperature at depth *z*, and $\Delta \dot{Q}\left(r_{5},z,t\right)$ is the heat rate transferred to the formation by the depth element Δz [5].

In 1962, Ramey proposed the following approximation for times longer than a week [5]:

(14) $$f\left(t\right)≅-\ln\frac{r_{5}}{2\sqrt{\alpha t}}-0.290$$

with α being the thermal diffusivity of the formation and *t* being the time measured in days.

In 1967, Matthews and Russell, assuming that the fluid inside the tubing could be considered a linear geometric heat source (line source) and given the small radius of the tubing compared to the radius of the formation, came up with the following approximation for the transient function [7]:

(15) $$f\left(\tau \right)≅\frac{1}{2}\int_{\frac{1}{4\tau }}^{\infty }\frac{e^{-u}}{u}du$$

where τ corresponds to the dimensionless Fourier time for formation, i.e.,

(16) $$\tau =\frac{k_{for}t}{\rho_{for}c_{for}{r_{5}}^{2}}$$

where $c_{for}$ is the specific heat of formation and $\rho_{for}$ is the mass density of the formation.

In 1994, Hasan and Kabir proposed the following approximation for the transient function referring to the condition of constant heat flow at the interface with the formation in $r_{1}$ [8,9]:

(17) $$\tau \leq 1.5\to f\left(\tau \right)=1.1281\sqrt{\tau }\left(1-0.3\sqrt{\tau }\right)$$

(18) $$\tau >1.5\to f\left(\tau \right)=\left[0.4063+0.5\ln\left(\tau \right)\right]\left(1+\frac{0.6}{\tau }\right)$$

In 2004, Hagoort proposed an approximate function for the constant temperature boundary condition at the internal interface of the formation. This is [10]:

(19) $$\tau <1\to f\left(\tau \right)=\frac{1}{\frac{1}{\pi \tau }+0.5-0.2\sqrt{\tau /\pi }}$$

(20) $$1<\tau <10^{5}\to f\left(\tau \right)=-0.0012{\left[\ln\left(\tau \right)\right]}^{3}+0.0249{\left[\ln\left(\tau \right)\right]}^{2}+0.3083\ln\left(\tau \right)+1.0504$$

(21) $$\tau >10^{5}\to f\left(\tau \right)=0.5\ln\left(\frac{\tau }{0.447}\right)$$

In general, the transient functions for boundary conditions of the “constant temperature at $r_{1}$”, “constant flow at $r_{1}$”, and “fluid with constant temperature and convection boundary condition at $r_{1}$” types are given, respectively, by

(22) $$\begin{array}{c} f\left(\tau \right)=1/L_{s\to \tau }^{-1}\{\frac{K_{1}\left(\sqrt{s}\right)}{\sqrt{s}K_{0}\left(\sqrt{s}\right)}\} \end{array}$$

(23) $$\begin{array}{c} f\left(\tau \right)=L_{s\to \tau }^{-1}\{\frac{K_{0}\left(\sqrt{s}\right)}{s\sqrt{s}K_{1}\left(\sqrt{s}\right)}\} \end{array}$$

(24) $$\begin{array}{c} f\left(\tau \right)=L_{s\to \tau }^{-1}\{\frac{1}{s}\frac{K_{0}\left(\sqrt{s}\right)}{K_{0}\left(\sqrt{s}\right)+\frac{\sqrt{s}}{\beta_{for}}K_{1}\left(\sqrt{s}\right)}\}/L_{s\to \tau }^{-1}\{\frac{1}{\sqrt{s}}\frac{K_{1}\left(\sqrt{s}\right)}{K_{0}\left(\sqrt{s}\right)+\frac{\sqrt{s}}{\beta_{for}}K_{1}\left(\sqrt{s}\right)}\} \end{array}$$

where $L_{s\to \tau }^{-1}\left\{-\right\}$ is the inverse Laplace transformation from *s* to τ, $K_{0}$ and $K_{1}$ are the Bessel functions of the second kind and order 0 and 1, respectively, and $\beta_{for}$ is the Biot number for the formation [6], i.e.,

(25) $$\beta_{for}=\frac{r_{1}U_{for}}{k_{for}}$$

where $U_{for}$ is the global heat transfer coefficient up to the interface with the formation, given by:

(26) $$\begin{array}{c} U_{for}={\left[\frac{1}{h}+\frac{r_{1}}{k_{tub}}\ln\left(\frac{r_{2}}{r_{1}}\right)+\frac{r_{1}}{r_{2}}\frac{1}{h_{comb}}+\frac{r_{1}}{k_{rev}}\ln\left(\frac{r_{4}}{r_{3}}\right)+\frac{r_{1}}{k_{cim}}ln\left(\frac{r_{5}}{r_{4}}\right)\right]}^{-1} \end{array}$$

The derivation of the listed equations, i.e., the solution of the system of differential equations for the mentioned boundary conditions, can be found in the work of Lima [4].

## 4. Analitical Approach

Wu and Pruess in 1990 [3], Assmann [11] in 1993, and Haggort in 2004 [10] presented analytical solutions for calculating the temperature of the fluid inside the tubing. Using the concept of the global heat transfer coefficient to synthesize the thermal resistances up to the formation, they all disregarded the transience in the completion. In addition, the work by Wu and Pruess presented data on the relationship between vertical and radial gradients, justifying the assumption of a zero vertical heat flow.

Although the solutions obtained by Wu and Pruess, Assmann, and Hagoort, among others, show good results after the well has been in operation for less than two weeks, like Ramey’s solution, disregarding the transience of the heat transfer in the completion, as well as the variation in the internal energy of the fluid (second part of Equation (10)), makes it impossible to obtain better results for times around the transit time of the fluid in the tubing.

**Figure 4:**
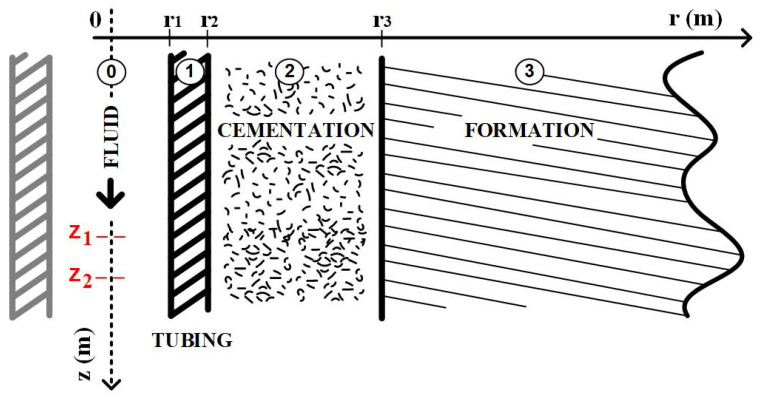
New control volumes and conservation of energy.

Consider the simplified completion shown in Figure 4, where the annulus has been ignored to illustrate the methodology. The layers of the well have been numbered to facilitate the naming of properties and variables. Furthermore, in the same figure, the chosen positions ($z_{1}$ and $z_{2}$) to calculate the integrals of Equation (10) are shown.

As we have seen so far, calculating the flow rate depends on knowing the value of the overall heat transfer coefficient, which in turn depends on the convection coefficient, the fluid temperature, the geothermal temperature, and the temperature of the internal tubing interface. Consider the following dimensionless temperatures for the fluid and the internal tubing interface:

(27) $$\theta_{f}\left(z,t\right)=\frac{T_{f}\left(z,t\right)-T_{g}\left(z\right)}{T_{g}\left(z\right)}$$

(28) $$\theta_{1}\left(z,t\right)=\frac{T\left(r_{1},z,t\right)-T_{g}\left(z\right)}{T_{g}\left(z\right)}$$

with $\theta_{f}$ being the injection fluid temperature and $\theta_{1}$ being the temperature of medium 1.

Since the heat flow by convection at the internal interface of the tubing must be equal to the heat flow at the same interface due to the use of the global heat coefficient, we have:

(29) $$U\left(z,t\right)=h\frac{T_{f}\left(z,t\right)-T\left(r_{1},z,t\right)}{T_{f}\left(z,t\right)-T_{g}\left(z\right)}$$

and, consequently:

(30) $$U\left(z,t\right)=h-\frac{\theta_{1}\left(z,t\right)}{\theta_{f}\left(z,t\right)}h$$

Considering that the vertical thermal gradient, and consequently the heat flow in the same direction, is only significant when compared to the intensity of the radial flow at the thermal disturbance front, which occurs during the fluid’s transit time in the tubing, it will be disregarded in this analysis [3]. Thus, excluding the vertical heat flow, assuming that the flow regime inside the tubing is turbulent, and applying the principles of conservation of energy and mass, we have the system of partial differential equations described in Table 1. The deduction of the equations and the solution can be found in Appendix A of the work by Lima [4].

As a result, the dimensionless temperature of fluid in the center of the tubing at r=0 and the dimensionless temperature at $r=r_{1}$ are given by:

(31) $$\begin{array}{c} \theta_{f}\left(w,\tau \right)=\frac{1}{T_{g}\left(w\right)}\left[-a_{w}L_{s\to \tau }^{-1}\{\frac{1-e^{-g(s)w}}{sg(s)}\}+b_{w}\theta_{f0}L_{s\to \tau }^{-1}\{\frac{e^{-g(s)w}}{s}\}\right] \end{array}$$

and

(32) $$\begin{array}{c} \theta_{1}\left(w,\tau \right)=\frac{1}{T_{g}\left(w\right)}\left[-a_{w}L_{s\to \tau }^{-1}\{{\bar{\bar{\theta }}}_{1}\left(w,s\right)\frac{1-e^{-g(s)w}}{sg(s)}\}+b_{w}\theta_{f0}L_{s\to \tau }^{-1}\{{\bar{\bar{\theta }}}_{1}\left(w,s\right)\frac{e^{-g(s)w}}{s}\}\right] \end{array}$$

where

(33) $$\begin{array}{c} g\left(s\right)=2\alpha_{1}\beta_{1}\left[1-{\bar{\bar{\theta }}}_{1}\left(w,s\right)\right]+s \end{array}$$

(34) $$\begin{array}{c} {\bar{\bar{\theta }}}_{1}\left(w,s\right)={\bar{c}}_{11}I_{o}\left(\sqrt{s}\right)-{\bar{c}}_{12}K_{o}\left(\sqrt{s}\right) \end{array}$$

such that:

(35) $$\begin{array}{c} {\bar{\theta }}_{1}\left(w,s\right)={\bar{\bar{\theta }}}_{1}\left(w,s\right){\bar{\theta }}_{f}\left(w,s\right) \end{array}$$

For Equations (31)–(35), ω is a dimensionless vertical coordinate, $a_{\omega }$ is the angular coefficient of geothermal temperature with respect to ω, $b_{\omega }$ is the linear coefficient of geothermal temperature with respect to ω, ${\bar{\theta }}_{1}$ is the dimensionless temperature of medium 1 in the Laplace domain, $\alpha_{1}$ is the thermal diffusivity of medium 1, and $\beta_{1}$ is the Biot number relative to medium 1.

The function g(s) is used to simplify the notation. The functions $I_{0}\left(\sqrt{s}\right)$ and $K_{0}\left(\sqrt{s}\right)$ are known in the literature as modified Bessel functions of the first and second kind, respectively, both of zero order. The variables ${\bar{c}}_{11}$ and ${\bar{c}}_{12}$ are functions in the Laplace domain, independent of the radial coordinate, *r*, and related to the *mean 1* of the completion under study, taken as a reference in the process of dimensioning the variables. Their values are given by a system of linear functions in the Laplace domain, which is a consequence of the boundary conditions of the problem. See [4] for more information.

In Equations (31) and (32), τ is the dimensionless time, *w* is the dimensionless vertical coordinate, $\theta_{f0}$ is the dimensionless temperature of the fluid at w=0, $a_{w}$ is the geothermal gradient related to the dimensionless coordinate *w*, and $b_{w}$ is the linear coefficient of the dimensionless geothermal temperature. The values of τ, *w*, $a_{w}$, and $b_{w}$ are given, respectively, by:

(36) $$\begin{array}{c} \tau =\frac{k_{1}}{\rho_{1}c_{1}{r_{1}}^{2}}t \end{array}$$

(37) $$\begin{array}{c} w=\frac{k_{1}}{v_{med}\rho_{1}c_{1}{r_{1}}^{2}}z \end{array}$$

(38) $$\begin{array}{c} a_{w}=\frac{v_{med}\rho_{1}c_{1}{r_{1}}^{2}}{k_{1}}a_{z} \end{array}$$

(39) $$\begin{array}{c} b_{w}=T_{g}\left(0\right) \end{array}$$

where $k_{1}$ is the thermal conductivity, $\rho_{1}$ is the mass density, $c_{1}$ is the specific heat related to medium 1, $v_{med}$ is the average velocity of the fluid velocity profile, and $a_{z}$ is the geothermal gradient related to independent variable z.

The dimensionless variables represented by Equations (27), (28), (36), and (37) are consequences of the algebraic manipulations that attempt to reduce the mathematical complexity of the system of partial differential equations. Among them, the dimensionless time variable (τ) is known in the literature as the dimensionless Fourier time.

Thus, in addition to Equation (30), the global heat coefficient can be rewritten as:

(40) $$U\left(\tilde{z},t\right)=h-\frac{-a_{w}L_{s\to \tau }^{-1}\{{\bar{\bar{\theta }}}_{1}\left(w,s\right)\frac{1-e^{-g(s)w}}{sg(s)}\}+b_{w}\theta_{f0}L_{s\to \tau }^{-1}\{{\bar{\bar{\theta }}}_{1}\left(w,s\right)\frac{e^{-g(s)w}}{s}\}}{-a_{w}L_{s\to \tau }^{-1}\{\frac{1-e^{-g(s)w}}{sg(s)}\}+b_{w}\theta_{f0}L_{s\to \tau }^{-1}\{\frac{e^{-g(s)w}}{s}\}}h$$

and, finally, the mass flow can be calculated by Equation (10), where the integral limits, $z_{1}$ and $z_{2}$, can be chosen in the interval belonging to the transport part of the tubing under analysis. $\tilde{z}$ can be assumed as an average of $z_{1}$ and $z_{2}$. In other words,

(41) $$\begin{array}{c} \tilde{z}≅\frac{z_{1}+z_{2}}{2} \end{array}$$

The inverse Laplace transforms of Equation (40) can be calculated numerically using the Gaver–Stehfest algorithm [12,13], which was adopted in this article, or other methods available in the literature.

As seen, it is possible to use a standard script for both approaches by continuing to use the global heat transfer coefficient as the basis for calculating the flow rate. However, unlike the previous quasi-static approach, the calculation of the global coefficient given by Equation (40) also considers the thermal transience in the layers of the well.

## 5. Computer Simulations

Given the very specific operating conditions regarding data collection and storage in the first moments of injection in a reservoir with the original initial conditions, the possibility of the existence of real, unmotivated data from the operation of a well in production under these specific conditions has proven unfeasible up to the point of the completion of this article, making it considerably difficult to evaluate the proposed analytical solution.

As a countermeasure to the lack of data for validation, a computerized well simulator was designed and developed to generate the temporal evolution of the thermal profile of the injection fluid along the tubing.

Due to its widespread use in the scientific community, the finite difference method, specifically the explicit Euler method, was used as a numerical tool in the simulator code development. In this method, the central idea is to replace the differential equations with algebraic equations, exchanging the derivatives for difference approximations and applying the resulting equations to each subdivision of the problem domain, referred to in this article as the control volume (CV). The result is an algebraic equation for each subdivision, making up a system of linear equations [6].

The code was developed in Phyton, version 3.9.13, from Anaconda Navigator, version 2.3.1. The code and packets used can be found in the work of Lima [4].

## 6. Results

Two computer simulations were carried out to evaluate the proposed method. The first simulation aimed to verify the alignment of the proposed solution with the classical quasi-static solutions proposed by Ramey and their successors. To this end, a schematic of a well without completion was adopted, aligning it with the reference model used to calculate the transient functions. The second simulation aimed to verify the proposed analytical solution in a well with simplified completion (tubing and cementing) and compare its performance with quasi-static solutions.

### 6.1. Simulation 1: Well without Completion

**Figure 5:**
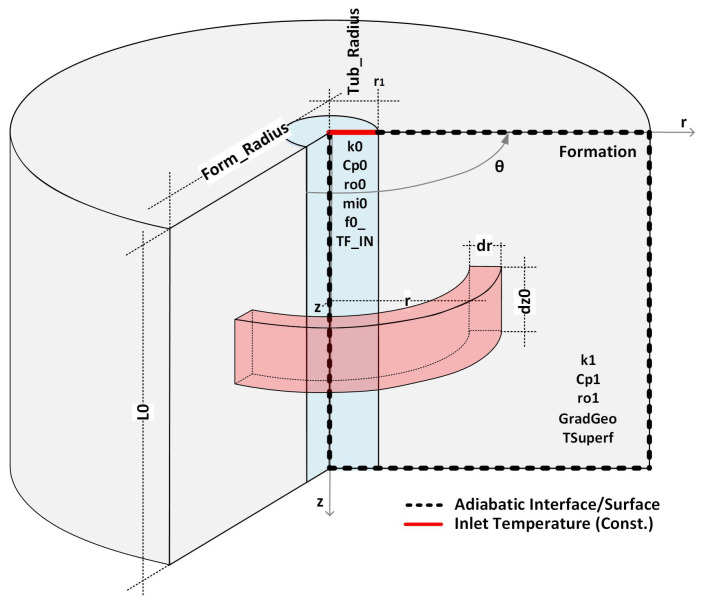
Well without completion—details of the problem.

In this first analysis, the data generated in the simulation of a well without completion were processed, as shown in Figure 5. This configuration is a good representation of the prototype well installed at UFRN [14]. Although the prototype has tubing, its high thermal conductivity means that its presence does not make a noticeable difference to the fluid temperature evolution, as will be seen below.

Once the oil industry has no wells without completions, the well shown in Figure 5 cannot be used as a real-world model. However, by processing the fluid temperature evolution for this configuration, it is possible to evaluate the contribution of the transient functions to the flow measurement since these functions were derived from a similar schematic of the well. Furthermore, since water injection wells operate for months, sometimes years, heat conduction in the formation is the dominant thermal process in flow measurements. Under these conditions, after the first two weeks of operation, any thermal transient in the completion that could influence the flow inference becomes insignificant [5]. In this way, a simulation without completion provides indications and trends for the behavior of the flow to be measured.

### 6.2. Simulation 1: Simulation Data

Table 2 shows the variables and values used in the simulation of the well shown in Figure 5. The other variables used in the simulation are functions of the values shown in the table.

### 6.3. Simulation 1: General Results

**Figure 6:**
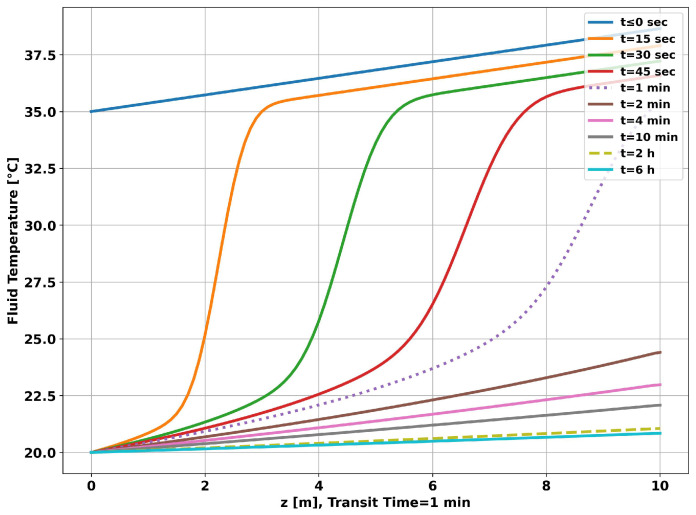
Well without completion—longitudinal temperature evolution.

Figure 6 shows the time evolution of the fluid temperature along the tubing. The curve for “t≤0 s” corresponds to the geothermal temperature. It is the initial condition used in solving the system of nonlinear equations. The curve for “t=1 min” corresponds to the temperature profile established after a transit time, which is the time required for the fluid to travel the total simulated 10 m. It can be seen that the dynamic change in the curve slows down as the operating time increases. In particular, the curve for 2 h differs little from the curve for 6 h.

**Figure 7:**
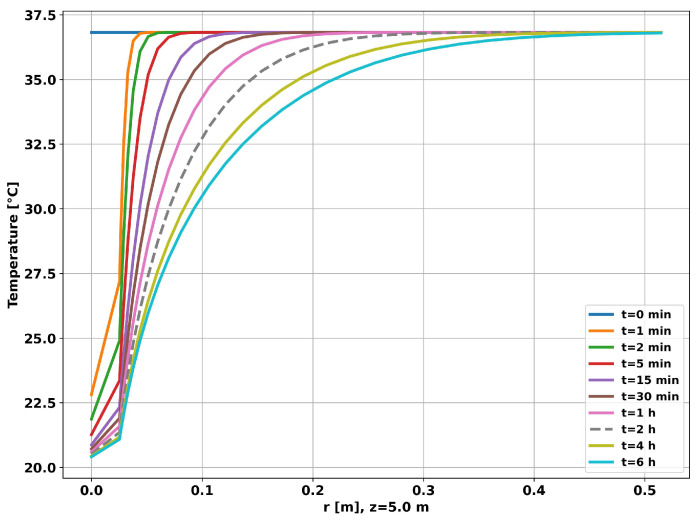
Well without completion—radial temperature evolution.

Figure 7 shows the time evolution of the radial temperature at a depth of 5 m. As can be seen in the graph, after 6 h, the thermal disturbance was approximately restricted to a distance of 50 cm from the center of the tubing, validating the external radius of the reservoir used in the simulation (1 m). Given the simulation time considered (6 h), using a small value for the outer radius of the reservoir (Reserv_Radius) would result in overheating/undercooling of the well, saturating the simulation and making the generated data unrepresentative of the real situation.

Therefore, for the prototype installed at UFRN, which has an outer radius of approximately 30 cm, we recommend adopting 2 h as the maximum operating time. After this time, the prototype will overheat, and the generated data will no longer be valid.

### 6.4. Simulation 1: Inferred Flow

**Figure 8:**
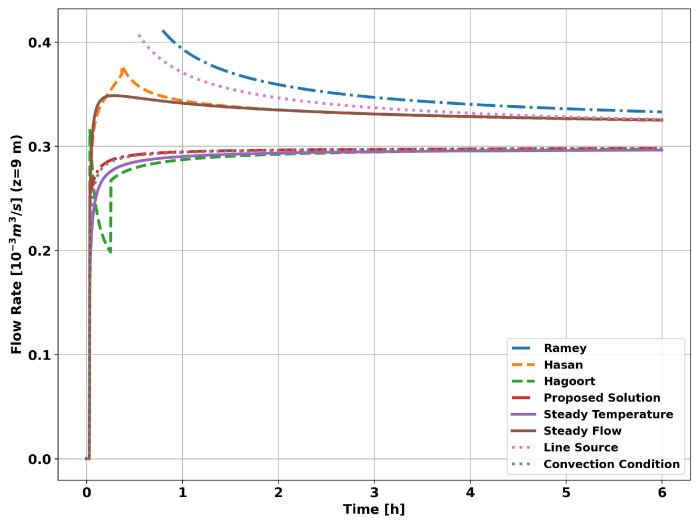
Well without completion—measurement at 9 m.

The graph in Figure 8 shows the evolution of the flow measurement for the methods and functions presented in this work. As can be seen, all the methods tend to converge to the reference flow used in the simulation (0.3 × 10${}^{-3}$ (m${}^{3}$/s)). However, the methods derived from the constant temperature assumption show better results, i.e., a constant temperature at $r_{1}$ (curve Constant Temp), a constant fluid temperature with convection conditions at $r_{1}$ (curve Convection Condition) and the Hagoort approximations (curve Hagoort). This greater accuracy of the constant temperature methods is a consequence of the short transit time of the fluid in the tubing, equal to 1 min, which means that the fluid temperature at the measurement point has a small time variation compared to the time variation of the heat flow.

**Figure 9:**
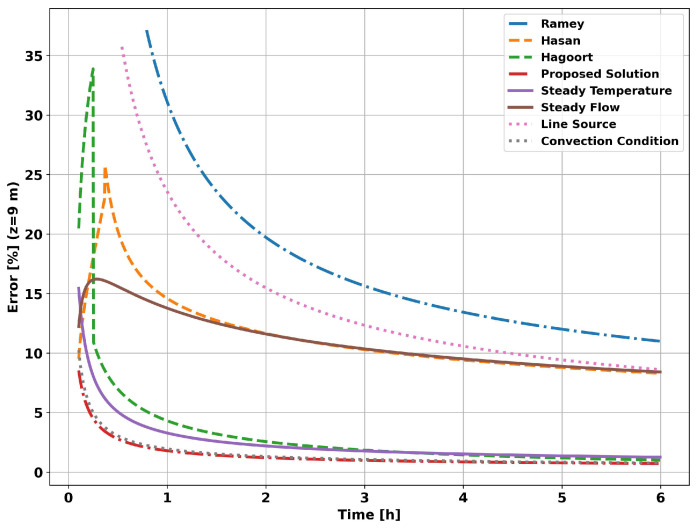
Well without completion—measurement error at 9 m.

The graph in Figure 9 shows the evolution of the flow measurement error. As expected, the choice of transient function will result in a greater or lesser systematic error. Applying the proposed analytical solution results in a significantly lower systematic error for the entire simulated operating time.

### 6.5. Simulation 2: Well with Simplified Completion

**Figure 10:**
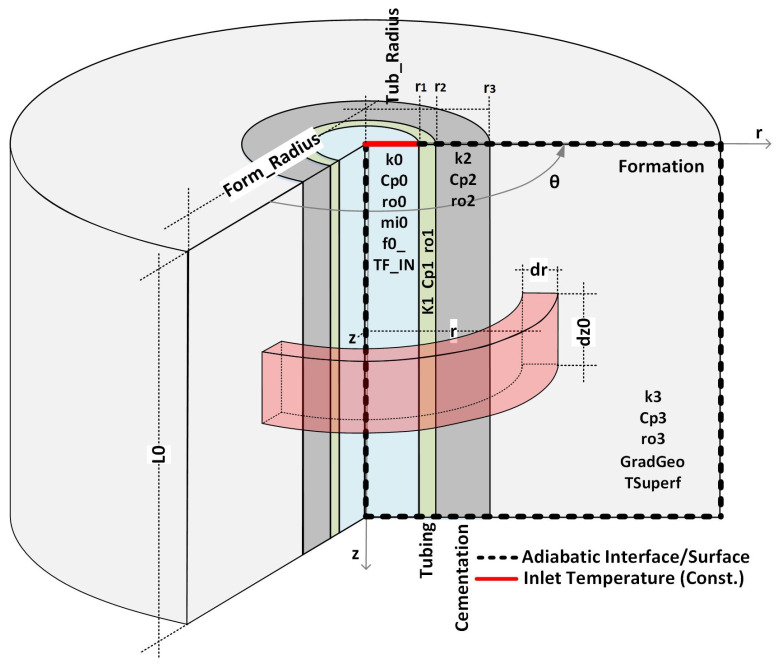
Completion with two layers—details of the problem.

This second simulation aims to verify the impact of transient heat transfer in the well completion on the inference of flow rate using the methods and formulations outlined in this article. To achieve this, consider the well completion represented in Figure 10. This completion technique, which involves only tubing and cementation, is widely utilized in real injector and producer wells.

As in the previous simulation, adiabatic boundaries were used at the edges of the simulated well, making it easy to detect saturation. A constant temperature was also used at the inlet of the injection column (tubing).

### 6.6. Simulation 2: Simulation Data

Table 3 shows the variables and values used in this simulation. Following the same approach as the previous simulation, the other variables used are functions of the values presented.

### 6.7. Simulation 2: General Results

**Figure 11:**
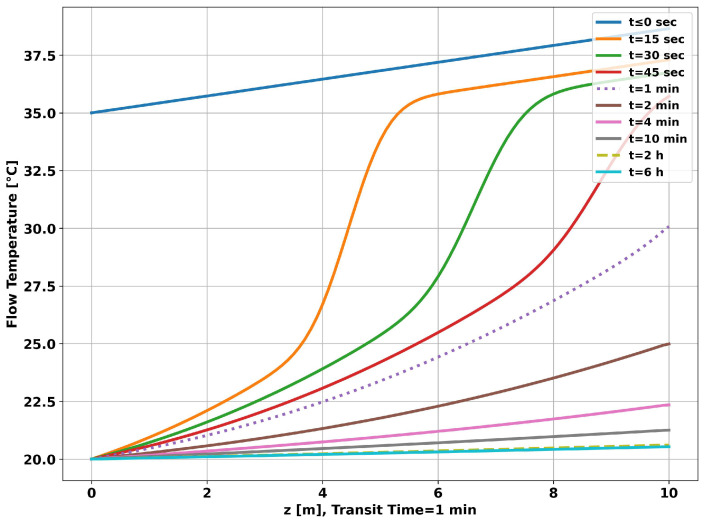
Completion with two layers—longitudinal temperature evolution.

The graph in Figure 11 shows the time evolution of the fluid temperature along the tubing. As in the previous simulation, the curve for “t≤0 s” corresponds to the geothermal temperature. It is the initial condition used in solving the system of nonlinear equations. It can be seen, as before, that the dynamics of the change in the curve lose speed as time progresses, but with less intensity, resulting in higher temperatures at the end of each measurement time. As observed in the previous simulation, the fluid temperature curve for 2 h of operation differs little from the curve for 6 h.

**Figure 12:**
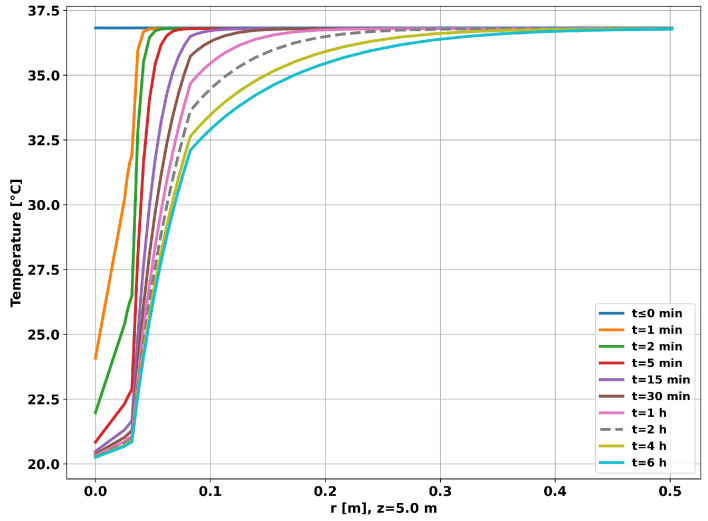
Completion with two layers—radial temperature evolution.

The graph in Figure 12 shows the time evolution of the radial temperature at a depth of 5 m, half the length of the tubing. After 6 h, the thermal disturbance was restricted to a distance of 50 cm from the center of the tubing, which shows that the simulation was not saturated for the time considered.

### 6.8. Simulation 2: Inferred Flow

**Figure 13:**
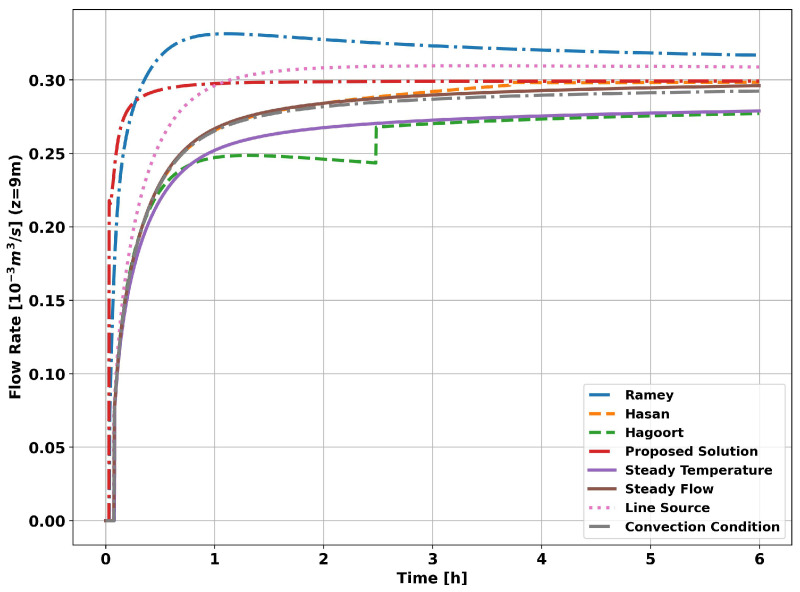
Completion with two layers—measured at 9 m.

When the flow is measured using the classical methods, simplifications are made in calculating the global heat transfer coefficient, i.e., the transient heat transfer through the completion (tubing and cementing) is not considered. When measured using the proposed analytical method, only the transient effects of the tubing are ignored due to its high thermal conductivity when compared to the other layers of the well (cementing and formation). The graph in Figure 13 shows the evolution of flow measurement for the methods and transient functions presented in this article.

As can be seen in Figure 13, as in the previous simulation, all the methods tended to converge to the reference flow rate (0.3 × 10${}^{-3}$ (m${}^{3}$/s)). However, unlike the previous simulation, the methods derived from the assumption of a constant heat flow, i.e., constant heat flow at $r_{1}$ (Constant Flow curve) and the Hasan and Kabir approximations (Hasan curve), showed better results. The method based on applying the convection boundary condition in conjunction with the assumption of a constant fluid temperature (Convection Condition curve) showed good results in both simulations, given its flexibility in dealing with heat flow into the reservoir. In the same sense, the proposed analytical solution (Proposed Solution curve) enabled flexibility in the fluid temperature and showed visibly better results than all the other methods studied.

**Figure 14:**
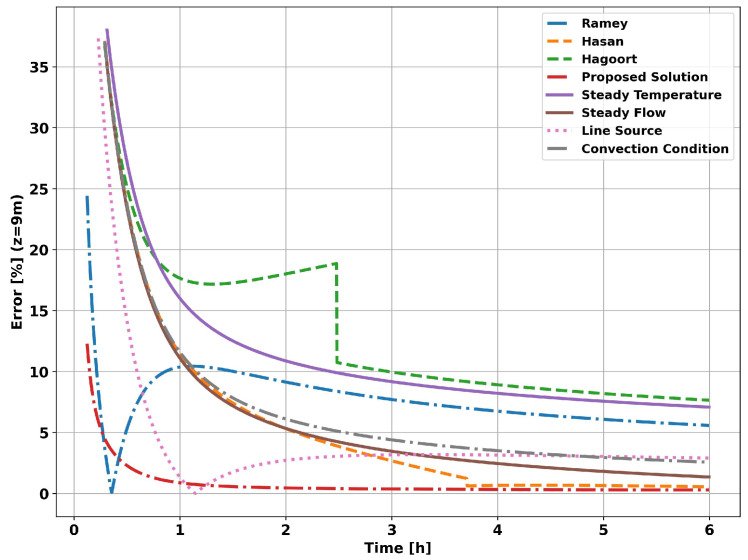
Completion with two layers—measurement error at 9 m.

The graph in Figure 13 shows the evolution of the systematic error in flow measurements. The systematic error resulting from applying the proposed analytical method (Proposed Solution curve) tends to be less than 1% in the initial moments of the simulation, unlike the other methods, which require hours or days of operation. The graph in Figure 14 shows the evolution of the systematic error in flow measurement. The systematic error in the initial instants of the analytical approach is a consequence of neglecting the heat flow in the vertical direction, which is significant in the initial moments in the vicinity of the well, and of not taking into account the effects of the tubing on heat propagation in the system as a whole.

## 7. Conclusions

This work proposed an analytical solution to predict the thermal profile of the fluid inside an injection well during the initial hours of well operation, a period not covered in the reviewed articles. The intention was to calculate the flow rate inside the tubing, crucial information for the success of an injection plan, and avoid damage to the oil reservoir. Unlike other studies, the transient heat flow in the well completion was addressed to achieve this, resulting in a complex system of partial differential equations.

Due to the absence of real data to evaluate the proposed solution, a computer simulation was conducted to predict the evolution of the thermal profile of the fluid inside the tubing. Two simulations were performed. The first, without any completion, was intended to verify the concordance of the proposed analytical solution with the solutions of Ramey and other researchers. The second, with a well-completion composed of tubing and cementation, was intended to verify the superiority of the proposed analytical solution.

Based on the data from the first simulation, it was observed that the proposed analytical solution is consistent with the quasi-static solution and shows slightly better results than the other studied solution over the six hours simulated. The error in the calculated flow rate was 1.8% after one hour and 1.2% after two hours of well operation. The average errors of the other solution studied were 13.2% and 9.2% after one and two hours, respectively. In the same way, using the data from the second simulation, the proposed analytical approach exhibits an error of 0.9% after one hour and an error of 0.5% after the first two hours, much less than the other solutions studied, which present an average error of 11.4% and 8.3% after one and two hours, respectively.

In summary, the proposed analytical solution has shown better results compared to the other solutions studied. Since the proposed solution considers the transient behavior of the heat flow in well completion, which, as was seen, is significant in the initial hours of well operation, these results were expected. However, the solution is more complex and more difficult to implement.

## Figures and Tables

**Table 1:** System of differential equations—simplified completion.

	Description	Equation
**Formation** **($r_{3}\leq r\leq \infty$)**	General Equation	$\frac{\partial^{2}T\left(r,z,t\right)}{\partial r^{2}}+\frac{1}{r}\frac{\partial T(r,z,t)}{\partial r}=\frac{1}{\alpha_{3}}\frac{\partial T(r,z,t)}{\partial t}$, $\;\alpha_{3}=\frac{k_{3}}{\rho_{3}c_{3}}$
	Initial Condition	$T\left(r,z,0\right)=T_{g}\left(z\right)$
	Boundary Condition (*∞*)	$\underset{r\to \infty }{lim}T\left(r,z,t\right)=T_{g}\left(z\right)$
	Boundary Condition ($r_{3}$)	(a) $k_{2}\frac{\partial T(r_{3}^{-},z,t)}{\partial r}=k_{3}\frac{\partial T(r_{3}^{+},z,t)}{\partial r}$
		(b) $T\left(r_{3}^{-},z,t\right)=T\left(r_{3}^{+},z,t\right)$
**Cementation** **($r_{2}\leq r\leq r_{3}$)**	General Equation	$\frac{\partial^{2}T\left(r,z,t\right)}{\partial r^{2}}+\frac{1}{r}\frac{\partial T(r,z,t)}{\partial r}=\frac{1}{\alpha_{2}}\frac{\partial T(r,z,t)}{\partial t}$, $\;\alpha_{2}=\frac{k_{2}}{\rho_{2}c_{2}}$
	Initial Condition	$T\left(r,z,0\right)=T_{g}\left(z\right)$
	Boundary Condition ($r_{3}$)	(a) $k_{2}\frac{\partial T(r_{3}^{-},z,t)}{\partial r}=k_{3}\frac{\partial T(r_{3}^{+},z,t)}{\partial r}$
		(b) $T\left(r_{3}^{-},z,t\right)=T\left(r_{3}^{+},z,t\right)$
	Boundary Condition ($r_{2}$)	(a) $k_{1}\frac{\partial T(r_{2}^{-},z,t)}{\partial r}=k_{2}\frac{\partial T(r_{2}^{+},z,t)}{\partial r}$
		(b) $T\left(r_{2}^{-},z,t\right)=T\left(r_{2}^{+},z,t\right)$
**Tubing** **($r_{1}\leq r\leq r_{2}$)**	General Equation	$\frac{\partial^{2}T\left(r,z,t\right)}{\partial r^{2}}+\frac{1}{r}\frac{\partial T(r,z,t)}{\partial r}=\frac{1}{\alpha_{1}}\frac{\partial T(r,z,t)}{\partial t}$, $\;\alpha_{1}=\frac{k_{1}}{\rho_{1}c_{1}}$
	Initial Condition	$T\left(r,z,0\right)=T_{g}\left(z\right)$
	Boundary Condition ($r_{2}$)	(a) $k_{1}\frac{\partial T(r_{2}^{-},z,t)}{\partial r}=k_{2}\frac{\partial T(r_{2}^{+},z,t)}{\partial r}$
		(b) $T\left(r_{2}^{-},z,t\right)=T\left(r_{2}^{+},z,t\right)$
	Boundary Condition ($r_{1}$)	(a) $-r_{1}^{+}\frac{\partial T(r_{1}^{+},z,t)}{\partial r}=\beta_{1}\left[T_{f}\left(z,t\right)-T\left(r_{1}^{-},z,t\right)\right]$, $\;\beta_{1}=\frac{r_{1}h}{k_{1}}$
		(b) $T\left(r_{1}^{-},z,t\right)=T\left(r_{1}^{+},z,t\right)$
**Fluid** **($0\leq r\leq r_{1}$)**	General Equation	$\frac{\partial T_{f}\left(z,t\right)}{\partial t}+v_{med}\frac{\partial T_{f}\left(z,t\right)}{\partial z}=\frac{2h}{r_{1}\rho_{0}c_{0}}\left[T\left(r_{1},z,t\right)-T_{f}\left(z,t\right)\right]$
	Initial Condition	$T_{f}\left(z,0\right)=T_{g}\left(z\right)$
	Boundary Condition ($z_{0}$)	$T_{f}\left(0,t\right)=T_{f0}$
	Boundary Condition ($r_{1}$)	(a) $-r_{1}^{+}\frac{\partial T(r_{1}^{+},z,t)}{\partial r}=\beta_{1}\left[T_{f}\left(z,t\right)-T\left(r_{1}^{-},z,t\right)\right]$, $\;\beta_{1}=\frac{r_{1}h}{k_{1}}$
		(b) $T\left(r_{1}^{-},z,t\right)=T\left(r_{1}^{+},z,t\right)$

**Table 2:** Well without completion—data used in the simulation (L = 10 m).

	Variable	Value	Description
**General data**	dt0	0.25 s	Time step
	dz0	0.1 m	Space step in the z direction (longitudinal)
	tsimu	6 h	Simulation time
	p_samples_z	0.1 m	Sampling period in the “z” direction
	p_samples_t	15 s	Time sampling period
	TAmb	35 ${}^{\circ }$C	Environment temperature
	TgeoType	1	Geothermal temperature type (0 = constant; 1 = linear)
	ThermalSource	0	0 = Adiabatic boundary; 1 = Thermal source equal
			to Tgeo
**Geometric**	L0	10 m	Longitudinal length of the well to be simulated
	Tub_Radius	0.0254 m	[r1] tubing inner radius
	Reserv_Radius	1 m	[r2] Reservoir external radius
	DIV0	1	Number of region radial divisions 0
	DIV1	40	Number of region radial divisions 1
**Fluid**	k0	0.636 W/(m·K)	Water thermal conductivity
	Cp0	4184 J/(kg·K)	Water-specific thermal capacity
	ro0	1000 kg/m${}^{3}$	Water-specific mass
	mi0	0.0006 N·s/m${}^{2}$	Absolute viscosity
	f0_	0.0003 m${}^{3}$/s	Volume flow
	TF_IN	20 ${}^{\circ }$C	Inlet fluid temperature
**Formation**	k1	2.42 W/(m·K)	Reservoir thermal conductivity
	Cp1	1500 J/(kg·K)	Reservoir-specific thermal capacity
	ro1	2100 kg/m${}^{3}$	Reservoir-specific mass
	GradGeo	0.365 ${}^{\circ }$C/m	Geothermal gradient in the “z” dimension
	TSurf	TAmb	Surface temperature

**Table 3:** Completion with two layers—data used in the simulation.

	Variable	Value	Description
**General data**	dt0	0.25 s	Time step
	dz0	0.1 m	Space step in the z direction (longitudinal)
	tsimu	6 h	Simulation time
	p_samples_z	0.1 m	Sampling period in the “z” direction
	p_samples_t	15 s	Time sampling period
	TAmb	35 ${}^{\circ }$C	Environment Temperature
	TgeoType	1	Geothermal temperature type (0 = constant; 1 = linear)
	ThermalSource	0	0 = Adiabatic boundary; 1 = Thermal source equal
			to Tgeo
**Geometric**	L0	10 m	Longitudinal length of the well to be simulated
	Tub_Radius	0.0254 m	[r1] Longitudinal length of the well to be simulated
	Reserv_Radius	1 m	[r4] Reservoir external radius
	DIV0	1	Number of region radial divisions 0
	DIV1	3	Number of region radial divisions 1
	DIV2	10	Number of region radial divisions 2
	DIV3	30	Number of region radial divisions 3
**Fluid**	k0	0.636 W/(m·K)	Water thermal conductivity
	Cp0	4184 J/(kg·K)	Water-specific heat capacity
	ro0	1000 kg/m${}^{3}$	Water-specific mass
	mi0	0.0006 N·s/m${}^{2}$	Absolute viscosity
	f0_	0.0003 m${}^{3}$/s	Volume flow
	TF_IN	20 ${}^{\circ }$C	Inlet fluid temperature
**Tub**	k1	14 W/(m·K)	Reservoir thermal conductivity
	Cp1	502 J/(kg·K)	Reservoir-specific thermal capacity
	ro1	8000 kg/m${}^{3}$	Reservoir-specific mass
	Tub_Thickness	0.635 cm	Tubing thickness
**Cement**	k2	0.9 W/(m·K)	Reservoir thermal conductivity
	Cp2	900 J/(kg·K)	Reservoir-specific thermal capacity
	ro2	2400 kg/m${}^{3}$	Reservoir-specific mass
	Cim_Thickness	5.08 cm	Cementation thickness
**Formation**	k3	2.42 W/(m·K)	Reservoir thermal capacity
	Cp3	1500 J/(kg·K)	Reservoir-specific thermal capacity
	ro3	2100 kg/m${}^{3}$	Reservoir-specific mass
	GradGeo	0.365 ${}^{\circ }$C/m	Geothermal gradient in the “z” dimension
	TSurf	TAmb	Surface temperature

## Data Availability

Data are contained within the article.

## Abbreviations

The following abbreviations are used in this manuscript:

α	Thermal diffusivity (m${}^{2}$/s)
$\alpha_{1}$	Thermal diffusivity of medium 1 (m${}^{2}$/s)
$\alpha_{2}$	Thermal diffusivity of medium 2 (m${}^{2}$/s)
$\alpha_{3}$	Thermal diffusivity of medium 3 (m${}^{2}$/s)
$\beta_{1}$	Biot number relative to the medium 1
$\beta_{for}$	Relative Biot number of the formation
μ	Absolute viscosity (N·s/m${}^{2}$)
$\theta_{1}$	Dimensionless temperature of the medium 1
${\bar{\theta }}_{1}$	Dimensionless temperature of medium 1 in the Laplace domain
$\theta_{f}$	Dimensionless injection fluid temperature
${\bar{\theta }}_{f}$	Dimensionless temperature of the injection fluid in the Laplace domain
rho ρ	Mass density (kg/m${}^{3}$)
$\rho_{0}$	Mass density of the medium 0 (kg/m${}^{3}$)
$\rho_{1}$	Mass density of the medium 1 (kg/m${}^{3}$)
$\rho_{2}$	Mass density of the medium 2 (kg/m${}^{3}$)
$\rho_{3}$	Mass density of the medium 3 (kg/m${}^{3}$)
$\rho_{for}$	Mass density of the formation (kg/m${}^{3}$)
τ	Shear stress (N/m${}^{2}$)
τ	Dimensionless Fourier time
$a_{w}$	Angular coefficient of geothermal temperature with respect to w (${}^{\circ }$C)
$b_{w}$	Linear coefficient of geothermal temperature with respect to w (${}^{\circ }$C)
*c*	Specific heat (J/(kg·K))
$c_{0}$	Specific heat of the medium 0 (J/(kg·K))
$c_{1}$	Specific heat of the medium 1 (J/(kg·K))
$c_{2}$	Specific heat of the medium 2 (J/(kg·K))
$c_{3}$	Specific heat of the medium 3 (J/(kg·K))
$c_{for}$	Specific heat of formation (J/(kg·K))
*f*	Friction factor
f(t)	Transient formation function
*h*	Heat convection coefficient (W/(m${}^{2}$·K))
$h_{comb}$	Combined heat transfer coefficient (W/(m${}^{2}$·K))
$I_{0}$	Modified Bessel function of first type and order 0
*k*	Thermal conductivity (W/(m·K))
$k_{0}$	Thermal conductivity of the medium 0 (W/(m·K))
$k_{1}$	Thermal conductivity of the medium 1 (W/(m·K))
$k_{2}$	Thermal conductivity of the medium 2 (W/(m·K))
$k_{3}$	Thermal conductivity of the medium 3 (W/(m·K))
$k_{cim}$	Thermal conductivity of cementation (W/(m·K))
$k_{for}$	Thermal conductivity of the formation (W/(m·K))
$k_{tub}$	tubing thermal conductivity (W/(m·K))
$k_{rev}$	Casing thermal conductivity (W/(m·K))
$K_{0}$	Modified Bessel function of second type and order 0
*L*	Length (depth) of the well (m)
$\dot{m}$	Mass flow (kg/s)
*r*	Radial coordinate (m)
$r_{1}$	tubing inner radius (m)
$r_{2}$	tubing external radius (m)
$r_{3}$	Casing inner radius (m)
$r_{4}$	Casing outer radius (m)
$r_{5}$	Cimentation external radius (m)
Re	Dimensionless Reynolds number
Pr	Prandtl dimensionless number
$\dot{Q}$	Heat transfer rate (W)
*s*	Transformation variable for the Laplace domain
*t*	Time (s)
*T*	Temperature (${}^{\circ }$C)
$T_{f}$	Average fluid temperature (${}^{\circ }$C)
$T_{g}$	Geothermal temperature (${}^{\circ }$C)
*U*	Overall heat transfer coefficient (W/(m${}^{2}$·K))
$\tilde{U}$	Average overall heat transfer coefficient (W/(m${}^{2}$·K))
$v_{med}$	Average velocity of the fluid velocity profile (m/s)
*w*	Dimensionless vertical coordinate
*z*	Vertical coordinate (m)
$z_{1}$	Initial vertical coordinate of the region under analysis (m)
$z_{2}$	Final vertical coordinate of the region under analysis (m)
$\tilde{z}$	Average vertical coordinate (m)
